# Increased Generation of TRAP Expressing Multinucleated Giant Cells in Patients with Granulomatosis with Polyangiitis

**DOI:** 10.1371/journal.pone.0042659

**Published:** 2012-08-08

**Authors:** Jin Kyun Park, Frederic Askin, Jon T. Giles, Marc K. Halushka, Antony Rosen, Stuart M. Levine

**Affiliations:** 1 Division of Rheumatology, Department of Medicine, Johns Hopkins University School of Medicine, Baltimore, Maryland, United States of America; 2 Department of Pathology, Johns Hopkins University School of Medicine, Baltimore, Maryland, United States of America; 3 Division of Rheumatology, Department of Medicine, Columbia University College of Physicians & Surgeons, New York, New York, United States of America; University of Michigan Medical School, United States of America

## Abstract

**Background:**

Tissue-infiltrating multinucleated giant cells (MNGs) within geographic necrosis are pathologic hallmarks of granulomatosis with polyangiitis (GPA). However, the origin, phenotype, and function of these cells in GPA remain undefined.

**Methodology/Principal Findings:**

MNG phenotype in GPA lung tissue was examined by immunohistochemistry using antibody directed against cathepsin K and calcitonin-receptor. Tartrate-resistant-acid-phosphatase (TRAP) expression was assessed using enzymatic color reaction. Peripheral blood mononuclear cells (PBMCs) from 13 GPA patients (5 with localized and 8 with systemic disease) and 11 healthy controls were cultured in the presence of RANKL and M-CSF for 9 days, and TRAP+ MNGs containing 3 or more nuclei were identified. GPA lung granulomata contained numerous MNGs that expressed osteoclastic TRAP and cathepsin K but not calcitonin receptors. In the presence of RANKL and M-CSF, PBMCs of GPA patients formed significantly more MNGs than healthy controls (114±29 MNG/well vs. 22±9 MNG/well, P = 0.02). In a subgroup analysis, patients with systemic disease generated significantly more MNGs than patients with localized disease (161±35 MNG/well vs. 39±27 MNG/well, P<0.01) or healthy controls (P<0.01). MNG production did not differ between localized GPA and control subjects (P = 0.96).

**Conclusions/Significance:**

MNGs in granulomata in the GPA lung express osteoclastic enzymes TRAP and cathepsin K. GPA patients have a higher propensity to form TRAP+ MNGs from peripheral blood than healthy controls. These data suggest that (i) the tendency to form MNGs is a component of the GPA phenotype itself, and (ii) that lesional MNGs might participate in the destructive process through their proteolytic enzymes.

## Introduction

Granulomatosis with polyangiitis (GPA), previously known as Wegener’s granulomatosis, is a systemic inflammatory disease whose phenotypic hallmarks include necrotizing vasculitis and granulomatous inflammation [1]–[3]. Although several infectious and environmental agents have been postulated to drive the inflammatory response in GPA, the properties of the tissue-infiltrating multinucleated giant cells in GPA remain unknown [4], [5].

Multinucleated giant cells (MNGs) can be found in both ostotic and non-ostotic sites [6]. Their ultimate function is highly dependent on the local cytokine environment; for instance, osteoclastic MNGs in bone form from macrophage precursors in the presence of macrophage colony stimulating factor (M-CSF) and receptor activator of NF-κB ligand (RANKL) and elaborate degradative enzymes such as cathepsin K and tartrate-resistant acid phosphatase (TRAP) that can digest bone mineral and matrix [7]–[10]. Conversely, in extra-ostotic granulomata, MNG formation is highly dependent on interactions with interleukin (IL) 4, 13, and 17, granulocyte-macrophage colony stimulating factor (GM-CSF) and interferon (IFN)-γ [11]. Much less is known about the properties of these peripheral MNGs, and the phenotype of extra-ostotic, tissue infiltrating MNGs in destructive GPA lesions has not been examined extensively to date. Because tissue-destructive granulomatous inflammation is a pathologic hallmark that separates GPA from other systemic inflammatory diseases [12], we asked whether GPA patients have an increased propensity to generate MNGs with tissue destructive enzymes from circulating precursors in the peripheral blood.

In this study, we demonstrate for the first time the presence of TRAP-expressing MNGs in lung granulomata and an increased propensity to form TRAP+ MNGs from circulating precursors in the peripheral blood in patients with GPA. This propensity appears to be associated with disease subtype rather than activity or immunosuppressive therapy.

## Results

### TRAP Expressing MNGs are Present within GPA Granulomata

**Figure 1:**
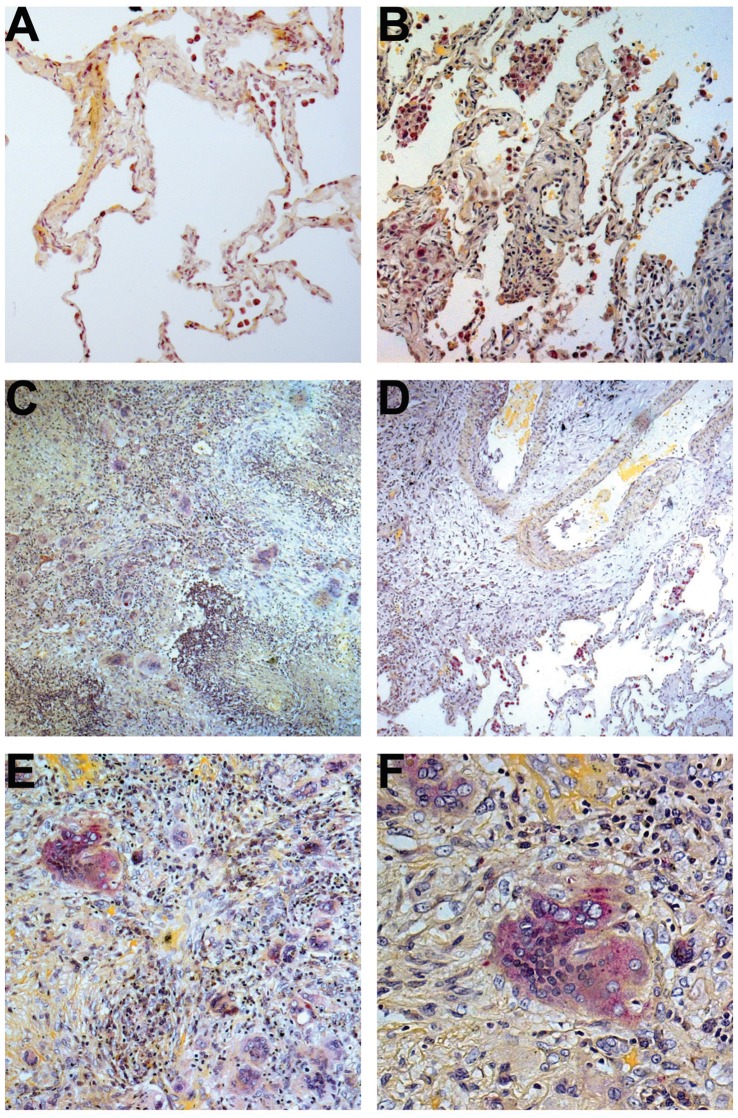
MNGs in the lung of patients with GPA express TRAP. Alveolar macrophages in control (A) and unaffected tissue in GPA lung (B) expressed TRAP (bright purple), and MNGs were not noted in normal lung tissue. A population of MNGs within the GPA granulomata expressed TRAP. TRAP expressing MNGs were localized in close vicinity to geographic necrosis (C) while MNGs were rarely seen in regions with few inflammatory infiltrates (D). MNGs varied in size and in number of incorporated nuclei. Further, the expression of TRAP varied between MNGs (E and F). Original magnification X 5 for C and D; X 10 for A, B and E; X 20 for F, respectively.*, TRAP positive macrophages; arrows, MNG; arrow heads, geographic necrosis.

To evaluate the lineage and phenotype of infiltrating MNGs in the GPA granulomata, lung biopsies from GPA patients (N = 11) containing areas of geographic necrosis and granulomatous inflammation were examined for expression of TRAP, which is a key degradative enzyme of bone-resident osteoclastic MNGs or osteoclasts. Alveolar macrophages in healthy and GPA lung expressed TRAP (bright purple cytoplasmic staining). MNGs were seen in 10/11 (91%) biopsies studied. They were not seen in healthy alveolar spaces (Figure 1A, B). MNGs were located adjacent to an area of geographic necrosis marked by the presence of numerous pyknotic and karyorrhetic PMNs (Figure 1C, arrow heads) while MNGs were rarely seen in areas of the lung containing few inflammatory infiltrates (Figure 1D). MNGs varied in size and number of incorporated nuclei. A population of MNGs within the GPA granulomata expressed TRAP. The TRAP+ MNGs were seen in 6/11 (55%) of the biopsies evaluated. Intensity of TRAP expression varied between the individual MNGs (Figure 1E, F).

**Figure 2:**
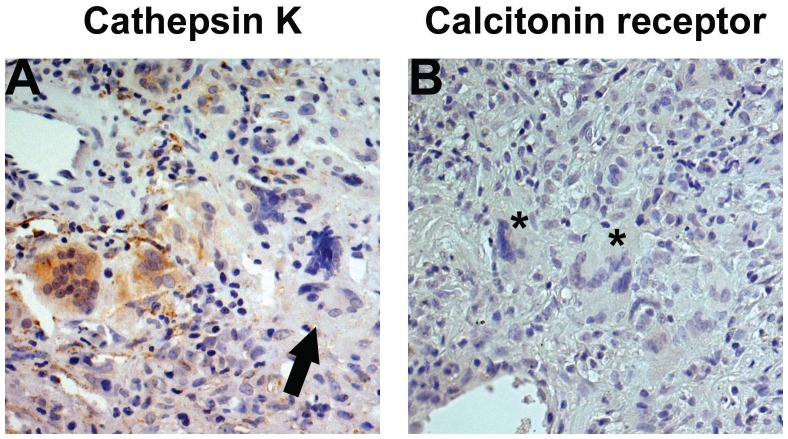
MNGs express cathepsin K. MNGs were evaluated for the expression of phenotypic markers of osteoclast cathepsin K and calcitonin-receptor using immunohistochemistry. The slides were counter-stained with hematoxylin. MNGs in the GPA granulomata expressed cathepsin K (A, brown cytoplasmic stain). However, it was not universally noted in all MNGs (A, arrow). MNGs (*) did not express calcitonin-receptors (B). Original magnification x 10.

We next investigated whether MNGs shared other critical markers of osteoclasts including cathepsin K and calcitonin receptor. A subset of MNGs expressed cathepsin K (Figure 2A) and they were observed in 4 out of 7 (57.1%) of biopsies. The intensity of cathepsin K expression varied between MNGs. Bronchial epithelial cells GPA lung tissue and infiltrating inflammatory cells demonstrated granular staining for calcitonin receptor (data not shown). Interestingly, none of the MNGs in 6 examined lung biopsies expressed calcitonin-receptors (Figure 2B, *), revealing a striking phenotypic difference to bone-resorbing osteoclasts which are characterized by the strong expression of TRAP, cathepsin K and calcitonin-receptors [7], [8], [13].

### Peripheral Mononuclear Cells of GPA have a Higher Propensity to Generate TRAP+ MNGs

**Figure 3:**
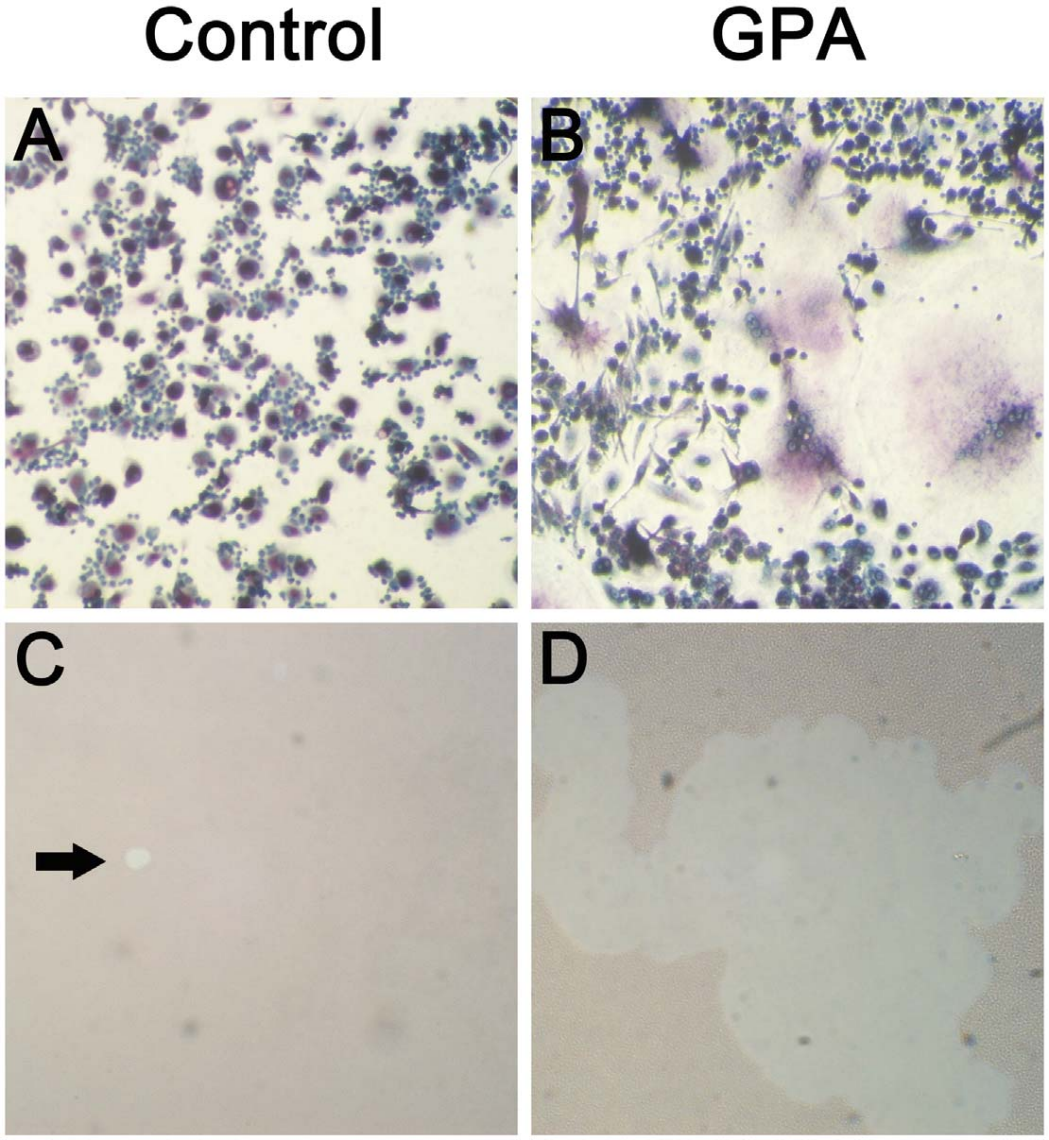
PBMCs from GPA patients form large TRAP+ MNGs and degrade bone matrix. PBMCs from both GPA patients and healthy controls were cultured in the presence of 25 ng/ml M-CSF and 100 ng/ml RANKL, and stained for the presence of TRAP expression after 9 days in culture. Nuclei were counterstained with hematoxylin. MNGs with three or more nuclei and TRAP expression (purple cytoplasmic stain) were counted. Few MNGs were generated at day 9 from the PBMCs of healthy controls (A); in contrast, large MNG with numerous nuclei were formed from patients with GPA (representative field shown in B). Further, the generated cells were able to degrade bone matrix. Numerous large pits were formed by GPA PBMCs (D) as compared to the healthy control PBMCs after 9 days (C). C and D are representative of 3 independent experiments. Original magnification x 10.

Given the presence of intra-lesional MNGs with TRAP expression in GPA lung, we explored whether patients with GPA have an increased propensity to form cells of this phenotype from circulating cells in the peripheral blood. In these experiments, PBMCs were isolated from 13 consecutive patients with GPA and 11 control subjects, and cultured in the presence of the OC-differentiating factors RANKL and M-CSF. After 9 days, PBMCs from GPA patients formed large TRAP+ MNGs with multiple nuclei. Further the generated cells were able to degrade bone matrix. In contrast, PBMCs from healthy controls formed fewer MNGs with smaller cytoplasm and had only minimal osteolytic activity (Figure 3). In addition, PBMCs from GPA patients generated more pits in-vitro than healthy controls (31.6±39.0/well vs. 2.6±3.1/well, P = 0.16), although this result was not statistically significant.

**Figure 4:**
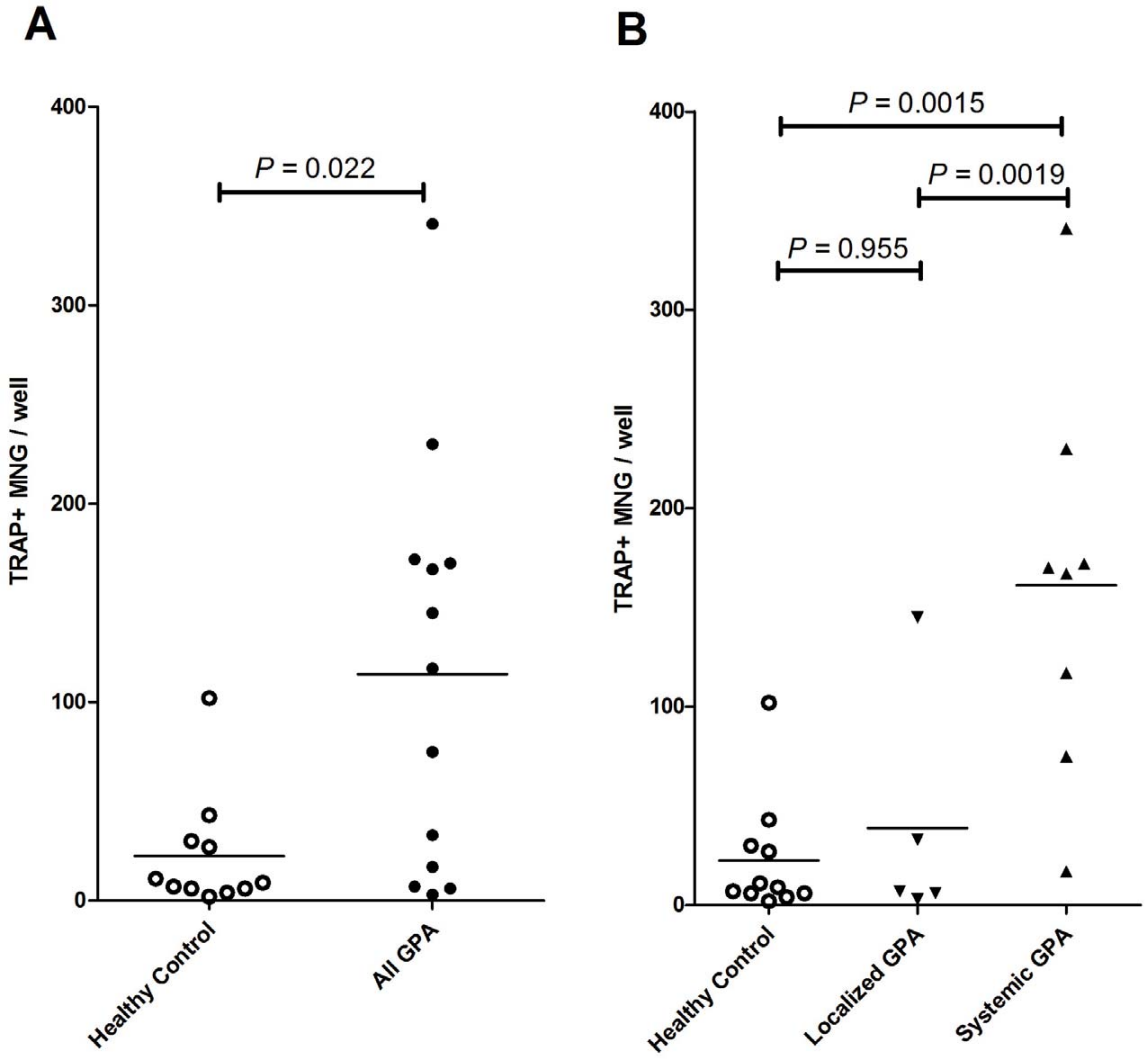
Increased MNG formation in patients with systemic GPA compared to those with limited disease. PBMC of healthy controls (N = 11) and GPA patients (N = 13) were cultured in presence of M-CSF and RANKL for 9 days and MNGs counted as in Materials and Methods. PBMCs from GPA patients generated significantly more TRAP+ MNGs than healthy controls (P = 0.022). When the GPA group was analyzed based on disease phenotype [ENT-localized (N = 5) vs. systemic form (N = 8)], only patients with systemic disease formed significantly more MNGs (P = 0.0015); no difference was seen between localized GPA and health controls (P = 0.955).

After 9 days, PBMCs from healthy controls formed 22.4±8.8 TRAP+ MNGs/well, while those from GPA patients formed 114.1±28.7/well (P = 0.022) (Figure 4A). Of note, only a few generated MNGs were TRAP negative. Interestingly, MNG formation in the GPA group seemed to have a bimodal distribution with high and low “MNG-formers”. We hypothesized that this bimodal distribution might track with distinct forms of the GPA phenotype. Based on the geographic extent of disease involvement, we separated the GPA cohort into two groups; one with a more limited geographic phenotype localized to the head and neck region, and a second with a more systemic form involving areas below the neck, including those with lung involvement. Of note, this grouping differs slightly from the Wegener’ Granulomatosis Etanercept Trial (WGET) definition of limited vs. severe GPA subtypes, in which limited GPA is defined by upper respiratory tract or lung manifestations with or without mild renal involvement [12]. In an analysis of TRAP+ MNG formation from PBMCs of patients within these subgroups, patients with systemic disease produced significantly more MNGs than did patients with localized disease (161±35 MNG/well vs. 39±27 MNG/well, P<0.01) or healthy controls (161±35 MNG/well vs. 22.4±8.8 MNG/well, P<0.01). There was no significant difference between control subjects and localized GPA (39±27 MNG/well vs. 22.4±8.8 MNG/well, P = 0.96) (Figure 4B).

### The Propensity to Form MNG is Associated with the GPA Phenotype

Given the heterogeneity of the patients evaluated in terms of disease activity and immunosuppressive medications (Table 1), we examined whether these parameters and the observed number of MNGs formed were related. As seen in Figure 4B, patients with systemic GPA generated more OC-like MNGs than those with a more limited phenotype. This difference could not be accounted for by differences between the groups based on disease activity measured by Birmingham Vasculitis Activity Score-Wegener’s Granulomatosis (BVAS-WG) (Spearman correlation, r_s_ = −0.1749, P = 0.57), or immunosuppressive medication regimen.

**Table 1:** Characteristics of GPA patients.

PatientCase #	Age, yrs	Sex	Duration, yrs	Location	ANCA Type	BVAS-WG	Mediation	TRAP+ MNG per well
1	27	F	5	systemic	c-ANCA	4	AZA/P	75
2	74	M	7	systemic	p-ANCA	2	RTX/P	17
3	51	F	5	localized	c-ANCA	2	AZA	145
4	46	F	3	localized	negative	1	AZA/P	33
5	64	M	3	systemic	c-ANCA	3	AZA	341
6	31	F	5	localized	c-ANCA	1	AZA/P	6
7	36	F	2	localized	c-ANCA	1	AZA/P	3
8	60	F	6	localized	c-ANCA	0	AZA/P	7
9	75	M	34	systemic	c-ANCA	0	AZA/P	172
10	72	M	2	systemic	negative	0	none	167
11	71	M	3	systemic	negative	0	AZA	230
12	47	F	3	systemic	c-ANCA	2	CTX/P	170
13	52	F	6	systemic	p-ANCA	2	RTX/MMP/P	117

Yrs, years; F, female; M, male; BVAS-WG, Birmingham Vasculitis Activity Score-Wegener’s Granulomatosis; TRAP, tartrate-resistant acid phosphatase; MNG, multi-nucleated giant cells; AZA, Azathioprine; P, Prednisone; RTX, Rituximab, CTX, cyclophosphamide; MMP, mycophenolate mofetil.

**Figure 5:**
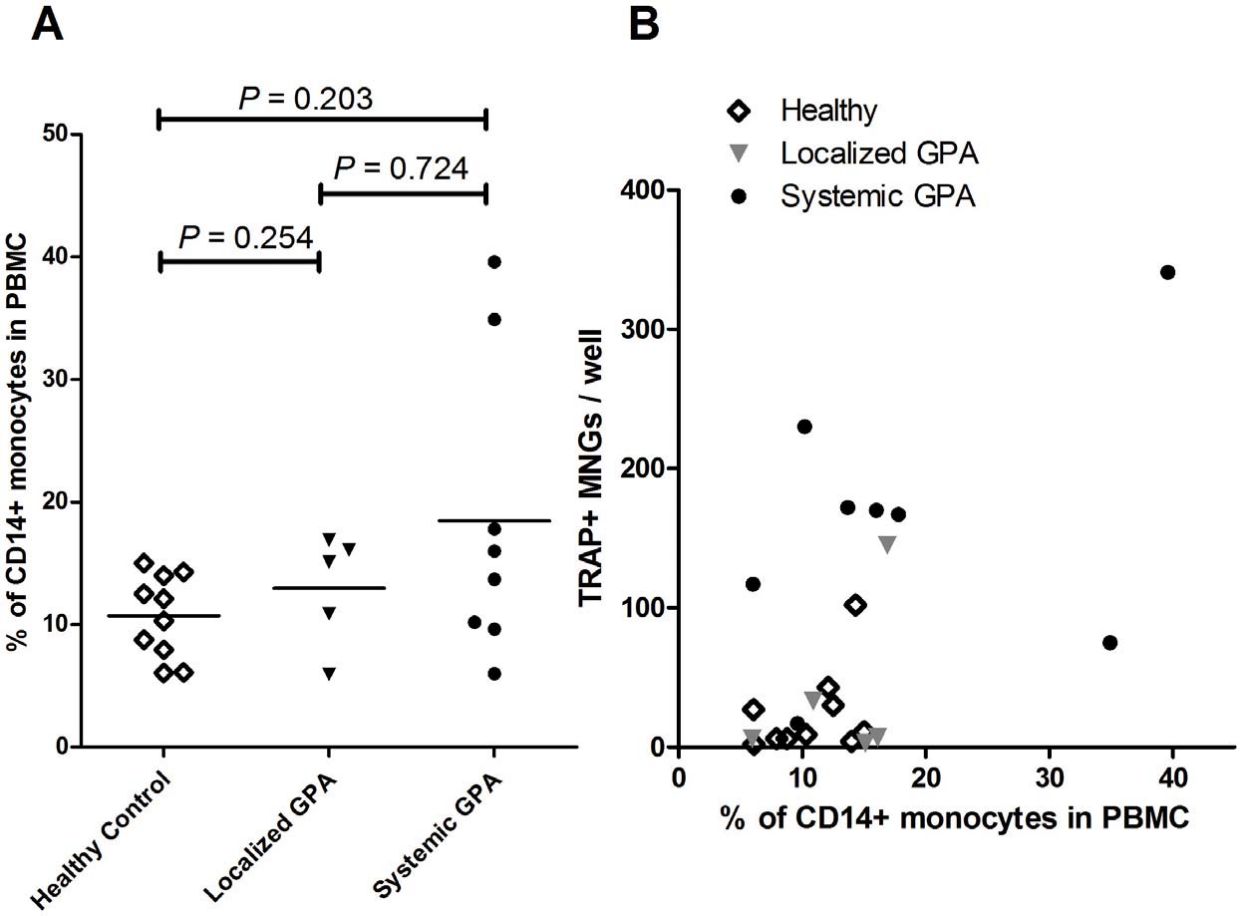
Lack of correlation is noted between MNG formation and circulating monocyte frequency in GPA. PBMC of healthy controls (N = 10), localized (N = 5) and systemic GPA (N = 8) were stained with anti-human CD14 antibodies and the percentage of CD14+ monocytes were determined using FACS. The number of the circulating monocytes did not differ between three groups (A). Further, percentage of CD14+ monocytes were plotted against formed MNGs per 2×10^5^ PBMC. There was no correlation between percentage of CD14+ monocytes in PBMCs and formed MNGs in both localized and systemic GPA (r^2^ = 0.19, P = 0.46 for localized GPA; r^2^ = 0.17, P = 0.30 for systemic GPA), indicating that systemic GPA formed higher number of MNGs per equivalent number of circulating CD14+ monocytes (B).

To address the possibility that the increased number of generated TRAP+ MNGs in GPA patients is a result of an increase in the total peripheral blood monocyte pool (where the MNG precursors might reside), the percentages of CD14+ monocytes were enumerated in each group by FACS. No significant difference was observed in the percentage of circulating monocytes between healthy controls and GPA patients (mean 10.7±1.1% vs. 16.3±2.8%; P = 0.129). Further, no significant differences in monocyte percentages were noted between patients with limited and systemic phenotypes (Figure 5A). Patients with systemic GPA generated higher numbers of TRAP+ MNGs than those with more localized disease and healthy controls with equivalent numbers of circulating CD14+ monocytes (Figure 5B). These data suggest that not the number, but the propensity of circulating monocytes to generate TRAP+ MNGs is increased in GPA patients presenting with the systemic phenotype.

## Discussion

A pathologic hallmark of granulomatosis with polyangiitis (GPA) is granulomatous inflammation with necrobiosis and tissue destruction. Despite the diagnostic specificity of this pathology, little is known about the lineage of the MNGs seen in this condition. In this study, we show for the first time that MNGs within the GPA lung granulomata express TRAP and cathepsin K, markers shared with osteoclasts. These specialized cells sharing biochemical features of bone-resorbing cells may contribute to the destructive lesions characteristic of this disease through the elaboration and release of proteolytic enzymes in the disease microenvironment [8], [10].

We also demonstrate that GPA patients have a higher propensity to form TRAP+ MNGs from the peripheral blood, and that this appears to be most pronounced in patients with more widespread disease manifestations. These data are consistent with those reported by De Costa and colleagues, who reported the presence of cathepsin K and TRAP-expressing MNGs in the destructive extra-ostotic lesions of Langerhans cell histocytosis [6]. Our study supports the notion that TRAP and cathepsin K expression is not limited to osteoclasts in bone and that MNG of this phenotype can arise in extra-ostotic sites in affected GPA tissue. Of special interest is the absence of calcitonin receptors, a key regulatory molecule of bone-resident osteoclasts. Their absence might suggest that in contrast to osteoclasts, “osteoclastic” activity of the lesional MNGs is uninhibited, contributing to continuous tissue damage. Currently, we are investigating whether the expression of TRAP and cathepsin K is a general feature of MNGs associated with granulomatous inflammation.

The observation that GPA patients have an increased propensity to form MNGs over healthy controls and that this difference is most prominent in patients with lung and systemic involvement is of great interest (Figure 4B). This difference was not associated with disease activity or treatment assignment. As GPA patients with localized phenotype rarely have granulomatous inflammation on sinonasal biopsies and a significant number of patients with this phenotype do not progress to a systemic phenotype [12], [14], [15], the ability to robustly form granulomata might be mechanistically relevant in generating this more systemic phenotype. The striking enrichment of MNG formation in patients with systemic disease is in contrast to our recent observation of a myelopoiesis gene expression signature in PBMCs, which was not associated with disease phenotype but with disease activity [16]. Furthermore, the propensity to form TRAP+ MNGs demonstrated in this study was not inhibited by immunosuppressive treatment despite good control of disease (as indicated by the low BVAS-WG scores seen in this cohort) as opposed to psoriatic arthritis where osteoclast precursors drop rapidly following treatment with anti-tumor necrosis factor (TNF) agents [17]. Therefore, this observation suggests that increased propensity of MNG formation might be a permanent intrinsic feature of systemic GPA regardless of disease activity and is not a result of ongoing systemic inflammation, which itself can increase the number of circulating osteoclast precursors [17]. Therefore, it would be crucial to know if a higher propensity of MNG formation normalizes when GPA goes into clinical remission. Further, understanding the mechanisms regulating MNG formation might provide insights into the pathogenesis of GPA.

While we have broadly defined that the circulating TRAP+ MNG precursor resides in the monocyte pool, the precise phenotype of these precursors is not yet defined. However, our findings cannot be explained by an increased number of circulating monocytes in GPA patients (Figure 4A). When isolated monocytes from healthy and GPA were cultured in the presence of M-CSF and RANKL, the numbers of generated MNGs were similarly high in both groups (data not shown). This finding suggests that monocytes in the presence of other regulatory cellular components including T cells of systemic GPA, and not CD14 cells *per se*, have a higher propensity to form TRAP+ MNGS. Recent work on osteoclastogenesis and foreign body giant cell formation indicates that the interactions between precursor cells, cytokines and growth factors, and tissues in the local microenvironment all contribute to a precise MNG differentiation program [11], [18], [19]. We propose that PBMCs in GPA have both the monocytes and additional cells/factors which make them “differentiation-competent” and accounts for their higher propensity to form TRAP+ MNGs.

Using finer phenotyping of the peripheral monocyte pool, Chiu and colleagues reported that the OC precursor may reside in the CD14+CD16+ monocyte compartment [20], and Mensah and colleagues have further characterized circulating OC precursors based on their surface expression of DC-STAMP and expression of other osteoclast differentiation markers [21]. Recently, Muto and colleagues described them as circulating cells that express high level of RANK and low c-Fms, receptor for M-CSF [22]. However, as lesional MNGs in GPA do not express any calcitonin receptor (figure 2B), MNGs in GPA and bone-resident osteoclasts might not share the same precursors within the CD14+ cellular compartment. Whether the TRAP and cathepsin expressing MNGs acquire their tissue degradative properties while still in the periphery or upon migration to the lung or other target tissue in the presence of local cytokines remains to be established; however, our data suggest that interfering in this MNG differentiation pathway therapeutically may provide a novel, directed approach at reducing GPA-related inflammation.

In summary, we demonstrate here for the first time the presence of TRAP and cathepsin K expressing MNGs in GPA lung granulomata and show that an increased propensity to form TRAP+ MNGs in circulating blood is a feature of generalized and not localized disease. These data suggest that the capacity to form MNGs might prove to be an important pathogenic biomarker in systemic disease and a possible novel therapeutic target in GPA.

## Materials and Methods

### Study Population

Thirteen consecutive GPA patients receiving clinical care in the Johns Hopkins Vasculitis Center and 11 control subjects in good health were enrolled in the study. Venous blood was collected from healthy volunteers under aseptic conditions. All subjects gave written informed consent, and all samples were obtained under the auspices of a Johns Hopkins University human subject institutional review board-approved protocol. All patients met both the 1990 American College of Rheumatology and 1994 Chapel Hill classification criteria for GPA [23]. Paraffin-embedded lung tissues were obtained from the Department of Pathology at the Johns Hopkins Medical Institutions. These tissues were originally obtained during the provision of clinical care. Separate informed consent to use these samples was not obtained- the general written informed consent form for surgery at the Johns Hopkins Medical Institutions contains language that gives permission for excess tissue not needed for diagnosis to be used for research purposes. All samples were provided without accompanying identifying demographic or clinical information to the authors under the auspices of a Johns Hopkins University institutional review board-approved protocol. This study was approved by the Johns Hopkins University institutional review of board.

### Cell Isolation

Peripheral blood mononuclear cells (PBMCs) were isolated from heparinized peripheral venous blood by density gradient centrifugation using Ficoll-Paque (GE Healthcare, NJ, USA). Cell viability was assessed with trypan blue dye exclusion.

### OC Culture and TRAP Assay

PBMCs (2×10^5^ per well) were cultured in OPTI-MEM® I (Gibco/Invitrogen, Grand Island, NY, USA), supplemented with 10% heat-inactivated fetal bovine serum, 1% penicillin and 1% streptomycin in the presence or absence of 100 ng/ml RANK-L (R&D Systems, Minneapolis, MN, USA) and 25 ng/ml M-CSF (R&D Systems, Minneapolis, MN, USA) in a 96 well plate in 5% carbon dioxide at 37°C. On day 9, cells were fixed with 3% formaldehyde and stained for expression of TRAP per the manufacturer’s protocol (Sigma, St Louis, MO, USA). TRAP+ cells with three or more nuclei were counted as TRAP+ MNGs under light microscopy Olympus CKX41 using magnification X 20. Numbers were expressed as mean ± SEM.

For pit formation assays, PBMCs were cultured in same condition as above in Corning® Osteo assay surface 96 well multiple plates (Corning Life Sciences, Tewksbury MA, USA) which are coated with inorganic crystalline calcium phosphate. After 9 days formed pits were counted using light microscopy.

### Immunohistochemistry

Formalin-fixed and paraffin- embedded tissues were de-parraffinized with xylenes and rehydrated. Antigen retrieval was performed at 95°C in citrate buffer (DAKO) and endogenous peroxidase was blocked with hydrogen peroxide (DAKO) for 10 minutes. After blocking, tissues were incubated with antibodies directed against cathepsin K (clone 3F9, Abcam, Cambridge, MA, USA) or calcitonin receptor (AHP635, AbD Serotec, Raleigh, NC, USA) overnight at 4°C. After washing in PBS, HRP conjugated secondary antibodies were applied for 1 hour at 37°C and the staining visualized with 3,3-diaminobenzidine (DAKO). Nuclei were counterstained with Meyer’s hematoxylin.

### TRAP Assay

After tissues were de-paraffinized and dehydrated as above, TRAP assay was performed using Leukocyte Acid Phosphatase Kit for TRAP per manufacture’s instruction (Sigma, St Louis, MO, USA). Nuclei were counterstained with Meyer’s hematoxylin. TRAP converts yellow color of substrate into bright pink.

### FACS Staining

PBMCs were incubated with anti-human CD14 PerCP (BD Biosciences, San Jose, CA, USA) and anti-human CD3 (BD Biosciences, San Jose, CA, USA) antibodies for 30 minutes at 4°C in the dark. Cells were washed with PBS and analyzed on a FACSCalibur flow cytometer. Flow data were analyzed by FlowJo software version 8.8 (Treestar, Ashland, OR, USA).

### Statistical Analysis

Mann-Whitney tests for continuous variables were performed. Correlations between data were performed using Spearman correlation. P-values of <0.05 were considered statistically significant. Statistical analyses were computed in GraphPad Prism (La Jolla, CA, USA).
